# Persistence and risk factors of occult hepatitis B virus infections among antiretroviral therapy-naïve people living with HIV in Botswana

**DOI:** 10.3389/fmicb.2024.1342862

**Published:** 2024-05-09

**Authors:** Motswedi Anderson, Bonolo B. Phinius, Basetsana K. Phakedi, Mbatshi Mudanga, Lynnette N. Bhebhe, Girlie N. Tlhabano, Patience Motshosi, Tsholofelo Ratsoma, Kabo Baruti, Gorata Mpebe, Wonderful T. Choga, Richard Marlink, Dieter Glebe, Jason T. Blackard, Sikhulile Moyo, Anna Kramvis, Simani Gaseitsiwe

**Affiliations:** ^1^Research Laboratory, Botswana Harvard Health Partnership, Gaborone, Botswana; ^2^School of Allied Health Professions, Faculty of Health Sciences, University of Botswana, Gaborone, Botswana; ^3^Botswana – University of Maryland School of Medicine Health Initiative, Gaborone, Botswana; ^4^Department of Biological Sciences, Faculty of Science, University of Botswana, Gaborone, Botswana; ^5^Rutgers Global Health Institute, Rutgers University, New Brunswick, NJ, United States; ^6^Institute of Medical Virology, National Reference Centre for Hepatitis B Viruses and Hepatitis D Viruses, Justus Liebig University of Giessen, Giessen, Germany; ^7^Division of Digestive Diseases, University of Cincinnati College of Medicine, Cincinnati, OH, United States; ^8^Department of Immunology and Infectious Diseases, Harvard T.H. Chan School of Public Health, Boston, MA, United States; ^9^Division of Medical Virology, Faculty of Medicine and Health Sciences, University of Stellenbosch, Cape Town, South Africa; ^10^School of Health Systems and Public Health, University of Pretoria, Pretoria, South Africa; ^11^Hepatitis Virus Diversity Research Unit, Department of Internal Medicine, School of Clinical Medicine, University of the Witwatersrand, Johannesburg, South Africa

**Keywords:** hepatitis B virus, HBV, occult hepatitis B, OBI, incidence, HIV/HBV, hepatitis B surface antigen (HBsAg) negative

## Abstract

**Aim:**

This study aimed to determine the kinetics of occult hepatitis B virus infections (OBI) among people with HIV (PWH).

**Methods:**

The study used archived plasma samples from longitudinal HIV natural history studies. We identified new OBI cases and assessed risk factors for OBI using Cox proportional hazards regression analysis.

**Results:**

At baseline, 8 of 382 [(2.1%) (95% CI: 1.06–4.1)] samples tested positive for hepatitis B surface antigen (HBsAg^+^). Of the 374 HBsAg-negative samples, 76 had sufficient sample volume for HBV DNA screening. OBI positivity (OBI^+^) at baseline was reported in 11 of 76 [14.7 95% CI (8.3–24.1)] HBsAg-negative (HBsAg^−^) participants. Baseline HBsAg-negative samples with sufficient follow-up samples (*n* = 90) were used for analysis of newly identified OBI cases. Participants contributed 129.74 person-years to the study and were followed for a median of 1.02 years (IQR: 1.00–2.00). Cumulatively, there were 34 newly identified OBI cases from the 90 participants, at the rate of 26.2/100 person-years (95% CI: 18.7–36.7). Newly identified OBI cases were more common among men than women (61.1% vs. 31.9%) and among participants with CD4^+^ T-cell counts ≤450 cells/mL (*p*-value = 0.02). Most of the newly identified OBI cases [55.9% (19/34)] were possible reactivations as they were previously HBV core antibody positive.

**Conclusion:**

There was a high rate of newly identified OBI among young PWH in Botswana, especially in men and in participants with lower CD4^+^ T-cell counts. OBI screening in PWH should be considered because of the risk of transmission, possible reactivation, and risk factors for the development of chronic liver disease, including hepatocellular carcinoma.

## 1 Introduction

It is estimated that approximately 2.73 million people with human immunodeficiency virus (HIV) [PWH] worldwide are coinfected with hepatitis B virus (HBV), with 1.96 million residing in sub-Saharan Africa (71%) (WHO, 2023). HBV/HIV coinfection has been reported to have a worse disease outcome than either mono-infection (Pinchoff et al., 2016; Rajbhandari et al., 2016; Maponga et al., 2020). In a meta-analysis, the prevalence of hepatitis B surface antigen (HBsAg) in PWH was reported as 5.3, 10, 6.7, and 11.4% in America, Europe, sub-Saharan Africa, and the World Health Organization (WHO) Western Pacific region, respectively (Leumi et al., 2020). In Botswana, HBsAg prevalence in PWH ranges between 3.1 and 10.6% (Wester et al., 2006; Matthews et al., 2015; Anderson et al., 2016; Mbangiwa et al., 2018; Phinius et al., 2023).

The detection of the hepatitis B surface antigen (HBsAg) is used for the routine screening of HBV (Raimondo et al., 2019). However, occult hepatitis B virus infections (OBI) are missed when only HBsAg is evaluated. OBI is described as the detection of replication-competent HBV deoxyribonucleic acid (DNA) in the liver/blood in the absence of detectable HBsAg in the blood (Raimondo et al., 2019). OBI presents as either seropositive or negative (i.e., for hepatitis B core antibody [anti-HBc] and/or HBV surface antibody) (Ryan et al., 2017; Raimondo et al., 2019; Gherlan, 2022). Isolated anti-HBc is often used as a proxy for OBI (Raimondo et al., 2019; Gherlan, 2022). The clinical relevance of OBI has been demonstrated in several studies (Cheung et al., 2010; Candotti et al., 2019; Eilard et al., 2019; Lelie et al., 2021; Satake et al., 2023). HBV from participants with OBI can be transmitted through blood transfusions and solid organ transplantations (Cheung et al., 2010; Candotti et al., 2019; Eilard et al., 2019; Lelie et al., 2021; Satake et al., 2023). OBI is common among PWH (Ryan et al., 2017; Raimondo et al., 2019; Phinius et al., 2023) and has also been detected in patients with serious clinical conditions such as hepatocellular carcinoma (HCC) and cirrhosis (Kew et al., 2008; Coppola et al., 2016; Ndow et al., 2022). This highlights the importance of proper diagnosis of OBI in both symptomatic and asymptomatic individuals. OBI prevalence rates ranging from 0 to 89.5% have been reported across the world in different at-risk groups, although the rates cannot be compared directly because of differences in the sensitivity of tests used and in the testing algorithms (Yuen et al., 2010; Bell et al., 2012; Escobedo-Melendez et al., 2014; Vargas et al., 2016). Reactivation of OBI can result in acute HBV infection in immunocompromised participants (Zachou et al., 2013). Additionally, in Botswana, work from our research group consistently reported more OBI positivity compared to HBsAg positivity. OBI prevalence in Botswana ranges between 6.6 and 33%, whereas HBsAg prevalence ranges between 2.1 and 8% (Ryan et al., 2017; Mbangiwa et al., 2018; Phinius et al., 2023).

Several factors may contribute to OBI. These include coinfections with HIV and/or hepatitis C virus, differences in host immune response in addition to epigenetic mechanisms, presence of HBsAg/hepatitis B surface antibody (anti-HBs) complexes, methylation of the HBV DNA, viral mutations, resolved HBsAg-positive infections, and reactivations (Lada et al., 2006; Mphahlele et al., 2006; Vivekanandan et al., 2008; Mallet et al., 2011; Zachou et al., 2013; Powell et al., 2015, 2016; Mardian et al., 2017; Raimondo et al., 2019; Wang et al., 2021, 2023; Gherlan, 2022; Kramvis et al., 2022; Zhang et al., 2022).

HIV has been shown to accelerate HBV infection progression, but the impact of HBV on the natural course of HIV has not been fully elucidated (Corcorran and Kim, 2023). In people with HBV, HIV leads to increased mortality, increased HBV chronicity, high HBV DNA levels, and hepatitis B e antigen (HBeAg) positivity (Hoffmann and Thio, 2007; Pinchoff et al., 2016; Rajbhandari et al., 2016; Maponga et al., 2020). The impact of HBV on HIV is less clear as some studies have shown that coinfection with HBV has no impact on the immunological or virological response to antiretroviral therapy (ART) in PWH (Hoffmann et al., 2009; Demosthenes et al., 2019). Most of the studies on the impact of HBV/HIV coinfection in natural disease progression were carried out in the HBsAg-positive individuals as in the studies described above. In contrast, there is a paucity of data on OBI natural disease progression owing to the few longitudinal studies on OBI, especially in Africa, where different genotypes/subgenotypes of HBV and subtypes of HIV circulate (Amponsah-Dacosta et al., 2018; Singh et al., 2019). A study in China followed seven OBI-positive blood donors, and all were HBV DNA negative at 1-year follow-up, without an intervention (Ye et al., 2016). Another study in Italy followed HCV-positive individuals and showed an association between OBI/HCV coinfection and an increased risk of progression to cirrhosis, HCC, and decreased survival rates (Squadrito et al., 2013). Chen et al. did not observe any association between OBI and worse clinical outcomes (Chen et al., 2017). Ignoring OBI might make the goal of eliminating viral hepatitis unattainable as it is a potential source of HBV transmission, morbidity, and mortality (de Almeida and de Paula, 2022). Therefore, we aimed to determine the kinetics of OBI in treatment-naïve PWH in Botswana.

## 2 Methodology

### 2.1 Study design, population, and sample size

This was a retrospective longitudinal study of antiretroviral therapy (ART)-naïve adults with HIV from Botswana. Archived plasma samples from two HIV natural progression studies (Botsogo and Dikotlana)—which were conducted at the Botswana Harvard Health Partnership in Gaborone, Botswana—were utilized for this study. Botsogo enrolled 442 participants and followed them up for 4 years (2005–2009) to determine the natural HIV progression (Farahani et al., 2016). Dikotlana study enrolled 878 (219 randomized to receive placebo, + 219 participants randomized to receive multivitamins alone, + 220 randomized to receive Selenium alone, and +220 randomized to receive multivitamins plus selenium) and followed for at least 24 months from 2004 to 2009 to evaluate the effect of micronutrient supplementation on disease progression (Baum et al., 2013). All available samples from the Botsogo and placebo group in Dikotlana study were used. A total of 382 samples from the two cohorts were screened. Participant selection was based on the availability of stored samples. The entire cohort was ART-naïve at baseline and at all subsequent visits as they did not qualify for ART according to the Botswana HIV treatment guidelines at that time that required PWH to have a certain CD4+ T-cell count threshold or an AIDS-defining illness for them to qualify for ART. None of the participants had cancer during the entire study period.

#### 2.1.1 Ethical approval and consent

The study was approved by the ethics review committee of the University of Botswana and the Health Research Development Committee (HRDC) at the Botswana Ministry of Health [Ethics permit number: PPME 13/1811V(318)]. The study participants provided written informed consent. This study was performed in line with the principles of the Declaration of Helsinki.

### 2.2 Laboratory methods

#### 2.2.1 HBV screening

Participant plasma samples were screened for HBsAg using the enzyme-linked immunosorbent assay (ELISA) Murex HBsAg version 3 kits (Murex Biotech, Dartford, UK) with a lower limit of detection of 0.13 IU/mL according to the manufacturer’s data (WHO, 2014). The QIAsymphony DSP Virus/Pathogen Kit was used to extract total nucleic acids in 600 μl of all samples with sufficient sample volume using an automated platform—QIAsymphony—according to the manufacturer’s instructions and eluted in 60 μl of buffer (Qiagen, Hilden, Germany). All HBsAg-negative samples were screened for OBI using an in-house HBV qualitative real-time polymerase chain reaction (qPCR) assay adapted from Kramvis’s research group with a lower limit of detection (LoD) of ∼20 IU mL (Bell et al., 2012). The real-time assay was performed in duplicate, and discordant results were repeated. This lower LoD is comparable to commercial platforms at 10–20 IU/mL and other in-house assays (Motta et al., 2010). These laboratory tests were performed at 12-month (1-year) intervals. The samples were also screened for anti-HBc using MONOLISA Anti-HBc PLUS (Bio-Rad, Marnes-la-Coquette, Paris, France) according to the manufacturer’s instructions at yearly intervals. HBV-positive samples (HBsAg and/or OBI positive) were screened yearly for HBeAg and anti-HBc immunoglobulin M (IgM) using MONOLISA HBeAg-Ab PLUS Kit (Bio-Rad, Hercules, CA) and MONOLISA Anti-HBc PLUS (Bio-Rad, Marnes-la-Coquette, Paris, France) respectively according to manufacturer’s instructions. Botsogo HBsAg screening and first time point anti-HBc screening were done in a separate study also using Murex HBsAg version 3 kits (Murex Biotech, Dartford, UK) and MONOLISA Anti-HBc PLUS (Bio-Rad, Marnes-la-Coquette, Paris, France), respectively, according to manufacturer’s instructions as outlined above (Phinius et al., 2020). The remaining Botsogo screening tests were performed with the Dikotlana samples; hence, the two cohorts were screened for similar HBV markers using similar methods. We assessed liver injury using the non-invasive markers, aspartate aminotransferase (AST)-to-platelet ratio index (APRI), and FIB-4.^1^ The APRI score is equal to 100 × (AST/40)/platelet, whereas the FIB-4 value is calculated as age [years] × AST [IU/L]/√ [PLT [10^9^/L] × (ALT [IU/L])].

#### 2.2.2 Data analysis

We estimated the rate of newly identified OBI cases with a 95% confidence interval (CI). Follow-up time for each patient was calculated from the baseline date of enrollment to the exact visit date of the first OBI result for OBI-positive cases and to the last date of an available sample for those that remained OBI-negative. The Cox proportional regression method was used to estimate hazard ratios (sex, age, HIV viral load suppression [≤400 or > 400] copies/mL, CD4+ T-cell count [≤450 or > 450] cells/mL) as prior studies have suggested a cutoff of 450 cells/mL for ART initiation for an increased survival rate as compared with lower CD4+ T-cell counts (Jain and Deeks, 2010; Ntekim and Folasire, 2010; Stohr et al., 2013; Assoumou et al., 2015). Fisher’s exact or chi-squared tests were used to compare categorical data where appropriate, whereas Wilcoxon rank sum or Kruskal–Wallis tests were used to compare continuous variables. Stata version 18.0 (StataCorp LLC, College Station, Texas, USA) was used to conduct all statistical analysis. *p*-values less than 0.05 were considered statistically significant.

## 3 Results

### 3.1 HBsAg and HBV DNA screening results

**Figure 1:**
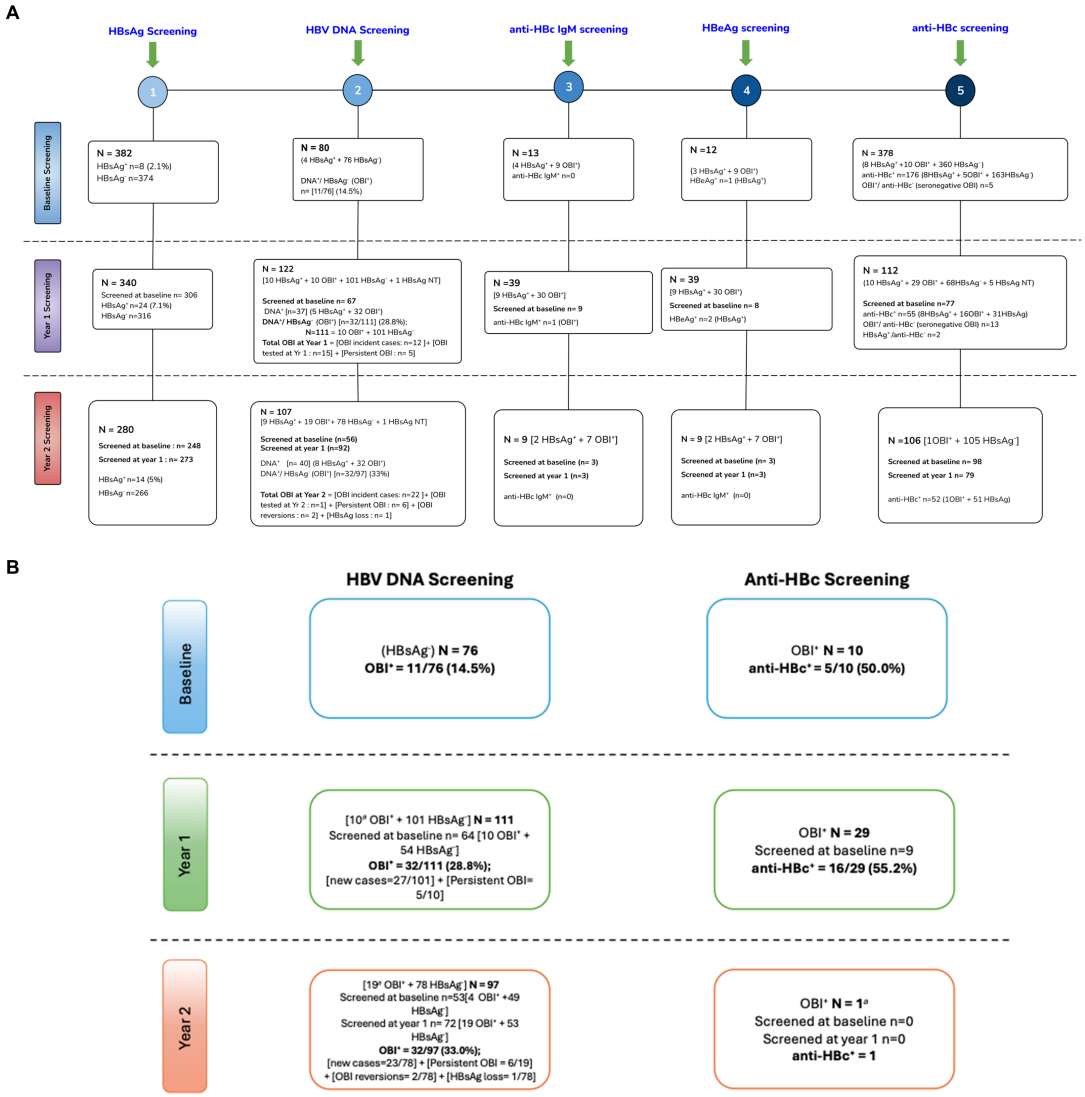
**(A)** Schematic flow chart of screening of HBV markers**. (B)** OBI kinetics. +, positive; −, negative; NT, not tested; anti-HBc, hepatitis B core antibody; HBeAg, hepatitis B e antigen; HBsAg, hepatitis B surface antigen; anti-HBc IgM, hepatitis B core antibody immunoglobulin M; OBI, occult hepatitis B infection; DNA, deoxyribonucleic acid; NB, some participants did not have available samples/sufficient volume for all tests. Some visits had more available samples than others.

At baseline, 8 of 382 [(2.1%) (95% CI: 1.06–4.1)] samples of PWH tested positive for HBsAg (HBsAg^+^). Of the 374 HBsAg-negative samples, only 76 had sufficient sample volume for HBV DNA screening. OBI positivity (OBI^+^) was reported in 11 of 76 [(14.7%) (95% CI: 8.3–24.1)] HBsAg-negative (HBsAg^−^) participants at baseline. At year 1, 340 participants were screened for HBsAg of which 306 were also screened at baseline; 24 of 340 [(7.1%) (95% CI: 4.8–10.3)] were HBsAg positive at year 1. Of the 340 participants, 122 [10 HBsAg^+^ + 111 HBsAg^−^ + 1 HBsAg not tested (NT)] had samples with sufficient volume for HBV DNA screening of which 67 were also screened at baseline. A total of 37 (5 HBsAg^+^ + 32 OBI^+^) samples were HBV DNA^+^, which gives HBV DNA^+^/ HBsAg^−^ (OBI^+^) rate of 32/111 [(28.8%) (95% CI: 21.2–37.9)] at year 1. Of the 122 participants screened for HBV DNA at year 1, there were 10 HBsAg^+^ and 32 OBI^+^ participants; hence, 32 of 42 (76%) cases with markers of active HBV infection were OBI. At year 2, 14 of 280 [(5%) (95% CI: 3–8.2)] participants who had available/sufficient sample tested positive for HBsAg, of which 248 and 273 were also screened at baseline and year 1, respectively. Of the 280 participants, 107 (9 HBsAg^+^ + 97 HBsAg^−^ + 1 HBsAg NT) had samples with sufficient volume for HBV DNA screening of which 56 and 92 were also screened at baseline and year 1, respectively. A total of 40 (8 HBsAg^+^ + 32 OBI^+^) samples were HBV DNA^+^, which gives OBI^+^ rate of 32 of 97 [(33%) (95% CI: 24.4–42.8)] at year 2. Of the 107 participants screened for HBV DNA at year 2, there were 9 HBsAg^+^ and 32 OBI^+^ participants; hence, 32 of 41 (78%) cases with markers of active HBV infection were OBI (Supplementary Figure S1; Figure 1A,B). There was no statistically significant difference between HBV-negative (HBsAg negative and OBI negative), HBsAg-positive, and OBI-positive participants for all variables tested at baseline except ALT. Participants with positive HBsAg serology had a significantly higher ALT level at baseline, although it should be noted that only four HBsAg+ participants had ALT data, Table 1.

**Table 1:** Participant demographics at baseline.

	**HBV negative** (HBsAg and OBI negative) ***n* = 65/84 (77.4%)**	**HBsAg positive** ***n* = 8/382 (2.1%)**	**OBI positive** ***n* = 11/76 (14.5%)**	***p*-value**
Sex, *n* (%), *n* = 84				
Female Male	51 (78.5) 14 (21.5)	5 (62.5) 3 (37.5)	10 (90.9) 1 (9.1)	0.3
Age, years, median (IQR) *n* = 84	33 (29–40)	31 (26–36)	34 (27–37)	0.6
CD4+ T-cell count, cells/mm^3^, median (IQR), *n* = 81	410 (317–511)	435 (305–631)	361 (279–477)	0.5
Log_10_ HIV VL, copies/mL, median (IQR), *n* = 82	4.16 (3.80–4.70)	4.13 (2.81–4.43)	4.15 (4.00–4.46)	0.6
ALT baseline, U/L, median (IQR) *n* = 79	15.1 (12.8–19.6)	23.4 (21.3–33.1)	15.0 (12.2–17.6)	0.04*
AST, U/L, median (IQR), (IQR), *n* = 83	23.2 (19.3–30.2)	30.9 (21.2–42.8)	21.2 (17.9–28.4)	0.3
PLT x 10^9^/L, median (IQR), *n* = 80	280 (244–327)	244 (212–332)	220 (214–303)	0.2
GGT baseline, IU/L, median (IQR), *n* = 69	16.2 (11.4–26.5)	24.3 (15.4–40.0)	17.2 (13.9–23.8)	0.6
APRI, median (IQR), *n* = 80	0.20 (0.17–0.27)	0.39 (0.15–0.42)	0.23 (0.19–0.32)	0.5
FIB4, median (IQR), *n* = 77	0.75 (0.58–0.90)	0.81 (0.50–1.41)	0.73 (0.61–0.96)	0.9

HBV, hepatitis B virus; HBsAg, hepatitis B surface antigen; OBI, occult hepatitis B infection; HIV, human immunodeficiency virus; VL, viral load; IQR, interquartile range; ALT, alanine aminotransferase; AST, aspartate aminotransferase; PLT, platelet; GGT, gamma-glutamyltransferase; APRI, AST-to-platelet ratio index; FIB-4, Fibrosis-4 index. NB, some participants have missing results; *Chi-squared *p*-values for categorical data and Kruskal–Wallis *p*-values for continuous variables.

### 3.2 HBsAg persistence/clearance

At baseline, there were eight HBsAg^+^ participants, all of whom were anti-HBc positive (anti-HBc^+^). The eight participants were retested at years 1 and 2; four participants remained HBsAg^+^ at both years 1 and year 2. At year 1, there were 24 HBsAg^+^ participants including 4 from baseline. Of the 20 newly identified HBsAg^+^ cases, 14 were incident cases as they were anti-HBc negative (anti-HBc^−^) at baseline. Only one was a possible reactivation as the participant was anti-HBc^+^ at baseline, whereas five participants could not be classified as they had no baseline anti-HBc results. Of the 24 HBsAg^+^ participants, 20 were tested again at year 2, and 7 (35%) remained HBsAg^+^. At year 2, 14 participants were HBsAg^+^ including 7 from year 1. Of the 7 newly identified HBsAg^+^ cases, all were incident cases as six were anti-HBc^−^ at baseline, whereas one participant was not tested for anti-HBc at baseline. However, that participant was anti-HBc^−^ at year 1.”

### 3.3 Anti-HBc results for OBI-positive participants

Participants with enough sample volume were further screened for anti-HBc as shown in Supplementary Figure S1 and Figures 1A,B. At baseline, there were 11 OBI^+^ participants, of whom 10 were tested for anti-HBc and 5/10 (50%) were anti-HBc^+^. At year 1, there were 32 OBI^+^ participants, of whom 29 had sufficient samples for anti-HBc screening and 16 of 29 (55.2%) were anti-HBc^+^ OBI. Of the 77 participants who were screened at both baseline and year 1, two participants lost the anti-HBc, whereas two also became anti-HBc^+^ at year 1. At year 2, there were 32 OBI^+^ participants of whom only one participant had sufficient volume for anti-HBc screening, and the participant was OBI anti-HBc negative (anti-HBc-). Anti-HBc^−^ OBI and anti-HBc^+^ OBI participants were compared. There was no difference in CD4+ T-cell count, HIV viral load, gender, age, AST, and ALT levels between anti-HBc^−^ OBI and anti-HBc^+^ OBI participants at baseline and year 1 (results not shown).

### 3.4 DNA clearance/persistence in plasma

Over time, several OBI^+^ cases persisted (remained OBI^+^ over time), whereas others tested OBI^−^ (HBV DNA-negative). This analysis was only possible for the OBI^+^ cases, who were tested at more than 1 time point. At year 1, 10 participants who were OBI^+^ at baseline were tested and 5 of 10 (50%) remained OBI^+^. There were also 12 OBI^+^ incident cases (participants with samples who were previously HBsAg^−^ and OBI^−^) and 15 OBI^+^ cases who were from participants being screened for OBI for the first time at year 1, resulting in a total of 32 OBI cases at year 1. At year 2, 19 participants who were OBI^+^ at year 1 were retested and 6 of 19 (31.6%) remained OBI^+^. Furthermore, 26 participants tested OBI^+^, of whom 22 were OBI incident cases, two were OBI reversions (were OBI^+^ at baseline, became OBI^−^ at year 1, and reverted to being OBI^+^ at year 2), one was due to HBsAg loss whereas another one was a participant being screened for the first time at year 2, giving a total of 32 OBI cases in year 2. Total new OBI cases over the 2-year period were 34, and one OBI case was because of HBsAg loss. Nineteen (55.9%) of the 34 new OBI cases were due to possible HBV reactivations as they were anti-HBc^+^ in previous visits. Two OBI^+^ participants, who had previously resolved OBI at year 1, reverted to OBI positivity at year 2, indicating intermittent OBI (Figure 1B).

### 3.5 Rate of newly identified OBI cases

The rate of newly identified OBI cases was subsequently analyzed, and only baseline HBsAg-negative samples with available follow-up samples were used for this analysis. This included HBsAg-negative samples, which did not have sufficient sample volume for DNA testing at baseline but had for subsequent visits. Testing was dependent on the availability of samples with sufficient volume for both HBsAg and HBV DNA screening. A total of 90 participants were followed up to estimate the rate of newly identified OBI cases over the entire follow-up period and approximately 80% (72/90) were women. Approximately 59% (43/73) of the participants had positive anti-HBc serology. Participant demographics were the same between those in the OBI estimation cohort and those who did not have available follow-up samples (Table 2). Participants contributed 129.74 person-years to the study and were followed for a median of 1.02 years (IQR: 1.00–2.00). Cumulatively, there were 34 newly identified OBI cases, giving a rate of 26.2/100 person-years (95% CI: 18.7–36.7). The median time to newly identified OBI was 367 days (IQR: 364–372).

**Table 2:** Demographics of participants used for OBI incidence estimation vs. the rest of the cohort.

	**Rest of cohort** ***n* = 305**	**Used for OBI estimation** ***n* = 90**	***p*-value**
Sex			
Female Male	245 (80.3) 60 (19.7)	72 (80) 18 (20)	0.5
Age, years	32 (28–39)	32 (29–40)	0.7
CD4+ T-cell count	456 (350–593)	416 (344–547)	0.2
Log HIV VL (IQR)	4.14 (3.44–4.67)	4.11 (3.70–4.72)	0.2
ALT bU/L (IQR)	17 (12–22)	15 (12–20)	0.5
AST U/L (IQR)	22 (18–28)	24 (19–30)	0.3
PLT x 10^9^/L (IQR)	255 (223–308)	276 (240–323)	0.05
GGT baseline, IU/L (IQR)	19 (14–33)	17 (13–26)	0.3
APRI (IQR)	0.21 (0.16–0.29)	0.20 (0.17–0.27)	0.9
FIB4 (IQR)	0.75 (0.61–0.93)	0.70 (0.56–0.88)	0.3

HBV, hepatitis B virus; HBsAg, hepatitis B surface antigen; OBI, occult hepatitis B infections; HIV, human immunodeficiency virus; VL, viral load; IQR, interquartile range; ALT, alanine aminotransferase; AST, aspartate aminotransferase; PLT, platelet; GGT, gamma-glutamyltransferase; APRI, AST-to-platelet ratio index; FIB-4, Fibrosis-4 index. NB, some participants have missing results.

**Figure 2:**
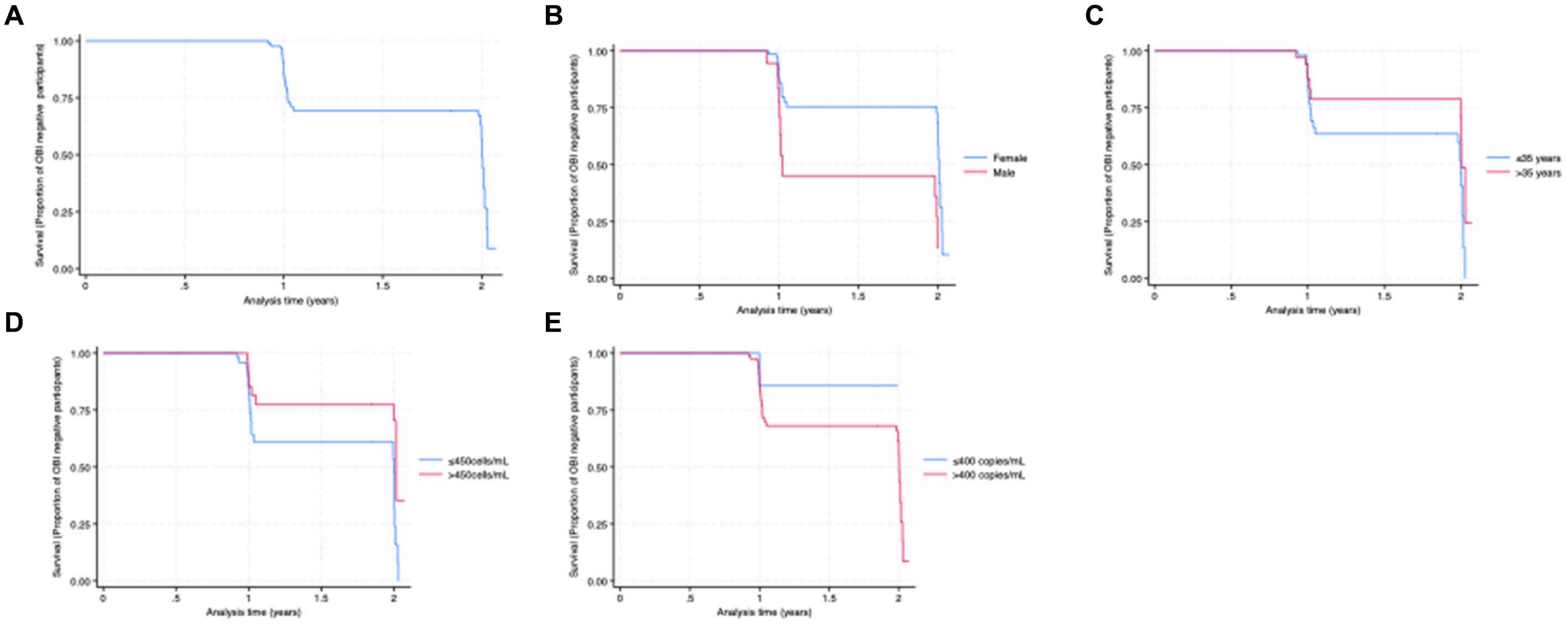
**(A)** Kaplan–Meier curve for the proportion of OBI survival (years). **(B)** OBI survival by sex. **(C)** OBI survival by age category. **(D)** OBI survival by CD4^+^ T-cell count category. **(E)** OBI survival by HIV viral load category.

Newly identified OBI cases were more frequent among men compared to women (61.1% vs. 31.9%, *p* = 0.02). Being male and CD4^+^ T-cell counts ≤450 cells/mL were associated with a significantly higher risk of newly identified OBI [hazard ratio 3.2 (95% CI: 1.5–6.8), *p*-value <0.01] and [hazard ratio 0.4 (95% CI: 0.2–0.9), *p*-value <0.02, respectively] (Table 3). Smooth hazard estimate results indicated that participants were more likely to experience the event (test OBI positive) after 1.5 years (Supplementary Figure S2). At 1 year of follow-up, 71% of the population remained OBI-negative (Figure 2A). Furthermore, there was a 57% chance that an individual remained OBI negative for more than 2 years. At the beginning of 1-year follow-up, 45% of men versus 75% of women tested negative for OBI (Figure 2B) and a higher number of older participants (>35 years) were OBI-negative compared to the younger group (<35 years) (Figure 2C). The probability of being OBI negative was higher among participants with higher CD4+ T-cell counts (>450 cells/ml) than those with lower CD4+ T-cell counts (Figure 2D), and OBI negativity was higher in participants with low HIV VL (< 400 copies/mL) than those with high VL (Figure 2E).

**Table 3:** Risk factors for OBI incidence.

**Variable**	**Incidence/100 person-years** **(95% CI)**	**Hazard ratio (95% CI)**	***p*-value**
Sex			
Female Male	21.7 (14.4–32.7) 48.0 (26.6–86.6)	Ref 3.2 (1.5–6.8)	<0.01
Age, years			
≤35 >35	31.2 (20.9–46.6) 19.3 (10.4–35.8)	Ref 0.5 (0.2–1.1)	0.1
HIV VL, copies/mL			
≤400 >400	7.9 (1.1–56.0) 28.4 (20.2–40.0)	Ref 2.3 (0.3–17.1)	0.4
CD4+ T-cell count, cells/mm3			
≤450 >450	35.1 (23.5–52.4) 15.9 (8.3–30.6)	Ref 0.4 (0.2–0.9)	0.02

HIV, human immunodeficiency virus; VL, viral load; CI, confidence intervals.

### 3.6 Impact of OBI on HIV disease progression

The impact of OBI on CD4+ T-cell decline and HIV VL over time was determined. There was no statistically significant difference in CD4+ T-cell count between incident OBI participants and OBI-negative participants at baseline, year 1, and year 2. Table 4. Incident OBI participants had a higher HIV VL than OBI-negative participants at year 1 (*p* = 0.02), (Supplementary Figure S3a); however, this difference was not observed with prevalent OBI participants at baseline and incident OBI participants in year 2, Table 4. After 1 year of follow-up, OBI-positive participants had an increased CD4+ T-cell count compared to a decrease in CD4+ T-cell count in OBI-negative participants (*p* = 0.01), Table 5 and Supplementary Figure S3b.

**Table 4:** Cross-sectional CD4+ T-cell count and HIV viral load at baseline, years 1 and 2.

**Outcome**	**OBI negative**	**OBI positive**	***p*-value**
CD4+ T-cell count (baseline), *n* = 72	406 (328–510)	361 (279–477)	0.3
CD4+ T-cell count (year 1), *n* = 88	441 (320–575)	358 (284–474)	0.1
CD4+ T-cell count (year 2), *n* = 71	414 (336–563)	415 (278–542)	0.5
Log_10_ HIV VL (baseline), *n* = 73	4.1 (3.7–4.7)	4.1 (4.0–4.5)	1.0
Log_10_ HIV VL (year 1), *n* = 89	4.0 (3.5–4.6)	4.5 (4.2–5.0)	0.02
Log_10_ HIV VL (year 1), *n* = 89	4.1 (3.4–4.8)	4.2 (3.9–4.7)	0.3

OBI, occult hepatitis B infections; HIV, human immunodeficiency virus; VL, viral load.

**Table 5:** HIV VL and CD4+ T-cell count change after 1 year of follow-up.

**Outcome**	**OBI negative**	**OBI positive**	***p*-value**
CD4+ T-cell count change (baseline to year 1), *n* = 72	−1 (−75–52)	72 (23–121)	0.01
CD4+ T-cell count change (year 1 to year 2), *n* = 70	−18 (−102–78)	32 (−101–6)	0.3
Log_10_ HIV VL change (baseline to year 1), *n* = 73	0.10 (−0.33–0.46)	0.13 (−0.04–0.26)	0.9
Log_10_ HIV VL change (year 1 to year 2), *n* = 89	0.00 (−0.40–0.57)	0.03 (−0.34–0.26)	0.6

OBI, occult hepatitis B infections; HIV, human immunodeficiency virus; VL, viral load.

### 3.7 HBeAg, anti-HBc IgM, and impact of anti-HBc on HIV disease progression

HBV-positive (HBsAg^+^ and/or DNA^+^) participants with enough sample volume were further screened for HBeAg and anti-HBc IgM at yearly time points. Anti-HBc IgM was performed to further confirm HBV incident cases and to explore its presence in OBI where it has not been extensively studied. At baseline, 12 (3 HBsAg^+^ + 9 OBI^+^) samples were screened for HBeAg, and 1/3 (33.3%) HBsAg^+^ participants were HBeAg positive (HBeAg^+^). At year 1, 39 (9 HBsAg^+^ + 30 OBI^+^) samples were screened for HBeAg and 2 of 9 (22.2%) HBsAg^+^ participants were HBeAg^+^. Of the 39 samples, 8 were also screened at baseline. At year 2, 9 (2 HBsAg^+^ + 7 OBI^+^) samples were screened for HBeAg, and none were HBeAg^+^. Of the nine samples, three were also screened at baseline and year 1.

At baseline, 13 (4 HBsAg^+^ + 9 OBI^+^) samples were screened for anti-HBc IgM and none were anti-HBc IgM positive (anti-HBc IgM ^+^). At year 1, 39 (9 HBsAg^+^ + 30 OBI^+^) samples were screened for anti-HBc IgM and 1/30 (3.3%) OBI^+^ participant was anti-HBc IgM^+^. Of the 39 samples, 9 were also screened at baseline. At year 2, 9 (2 HBsAg^+^ + 7 OBI^+^) samples were screened for anti-HBc IgM and none were anti-HBc IgM ^+^. Of the nine samples, three were also screened at baseline and year 1.

The impact of anti-HBc on CD4+ T cell and HIV viral load change over time was determined. Anti-HBc did not have an impact on CD4+ T-cell decline and HIV viral load increase over time (results not shown). Anti-HBc-positive participants had significantly higher CD4^+^ T-cell counts at year 1 than anti-HBc-negative participants (*p*-value = 0.03). Supplementary Table S1.

## 4 Discussion

OBI is a common phenomenon, especially among PWH, and has clinical relevance as HBV can be transmitted, which can lead to the possible development of liver disease including HCC (Saitta et al., 2022). Hence, neglecting OBI might compromise the goal of eliminating viral hepatitis by 2030 as OBI can be a reservoir for HBV transmission, morbidity, and mortality (de Almeida and de Paula, 2022). There are sparse data on the natural progression of OBI, especially in sub-Saharan Africa, owing to few longitudinal studies in the area; therefore, this study sought to close this gap. Studying the natural progression of OBI among PWH, a group mostly affected by OBI, is currently quite challenging in the HIV test-and-treat era because most of the antivirals used act against both HIV and HBV (WHO, 2023). We report here, in a natural HIV cohort, a high rate of newly identified OBI, 26.2/100 person-years with most (55.9%) newly identified OBI cases being participants who were previously HBsAg-negative and anti-HBc positive (possible reactivations) leading to seropositive OBI. We further report loss of anti-HBc leading to anti-HBc-newly identified OBI cases. The study participants were recruited before the “test and treat era” (2004–2009) and did not qualify for treatment according to the Botswana National HIV guidelines at that time which required PWH to have a certain CD4+ T-cell count threshold or an AIDS-defining symptom to qualify for treatment.

The high rate of newly identified OBI in this study was expected as OBI has previously been reported in PWH elsewhere (Terrault et al., 2018; Saitta et al., 2022). Furthermore, OBI prevalence rates in PWH in Botswana are high, indicating that this is a frequent phenomenon in the country (Ryan et al., 2017; Mbangiwa et al., 2018; Phinius et al., 2023). Indeed, high HBsAg incidence rates were reported by a previous study in Botswana (Phinius et al., 2020). Additionally, there was an increase in HBsAg-positive participants from baseline to year 1. This result is concordant with literature such as the Botsogo study (Phinius et al., 2020) and a study in South Africa (Msomi et al., 2020). This may be the result of decreasing CD4^+^ T-cell counts as shown previously (Phinius et al., 2020). Approximately 75% of HBV active cases were OBI. This is consistent with literature reported from Botswana, where there are consistently more OBI cases compared to HBsAg-positive cases in both HIV/HBV coinfection and in HIV-negative individuals (Ryan et al., 2017; Mbangiwa et al., 2018; Phinius et al., 2023). High OBI prevalence compared to HBsAg prevalence was also reported in South Africa (Amponsah-Dacosta et al., 2015) and India (Dinesha et al., 2018). In contrast, another Indian study reported more HBsAg compared to OBI (Saha et al., 2017). Sequencing was not performed in the current study, but differences might be due to the different circulating genotypes where the predominant subgenotype in Botswana and South Africa is A1 compared to D1 and D2 reported in the study from India. Mutations in the HBsAg may be responsible for the high OBI rate in Botswana (Powell et al., 2016; Anderson et al., 2018a,b). The role of HBV vaccination is assumed to be very minimal in this study as in Botswana HBV infant vaccination commenced approximately 2000 when the current study participants were at least adolescents (Patel et al., 2011). The varying sensitivity rate of assays used for OBI screening further leads to varying OBI frequency across studies making comparisons difficult.

In this study, most (55.9%) of the newly identified OBI cases had prior HBV exposure as shown by anti-HBc positivity and hence were possible HBV reactivations. HBV reactivations are a known cause of OBI (Terrault et al., 2018; Saitta et al., 2022). HBV reactivation is common following immunosuppression, and indeed in the present study, lower CD4^+^ T-cell counts were a risk factor for newly identified OBI. Only one newly identified OBI case was the result of HBsAg loss. This phenomenon is also supported by the literature (Saitta et al., 2022). A study in South Africa reported a higher percentage of HBsAg participants, who progressed to OBI during treatment (Amponsah-Dacosta et al., 2018). The kinetics of OBI in treatment-naive individuals may differ from those in treated individuals. Some anti-HBc-OBI cases were due to loss of anti-HBc, whereas others were anti-HBc-negative initially. This is also consistent with the literature (Saitta et al., 2022). Interestingly, of the newly identified HBsAg-positive cases, only one was due to possible reactivation indicating that in this study most possible reactivations resulted in OBI positivity and not HBsAg positivity, which is consistent with data from Botswana (Phinius et al., 2020). A study in Ethiopia reported a 14% prevalence of mutations associated with HBV reactivations in an OBI cohort (Patel et al., 2020). Such studies are warranted in Botswana as they might explain some of the possible reactivation seen in this study. Anti-HBc has been and is still used as a proxy for OBI. Our results which agree with the literature show that this screening algorithm misses OBI cases that are anti-HBc negative (Ryan et al., 2017; Kramvis et al., 2022; Saitta et al., 2022). DNA remains the only reliable marker for OBI diagnosis indicating a need in investing in serum markers that bridge the gap between the OBI as defined by anti-HBc vs. HBV DNA to simplify HBV diagnosis and improve patient care.

Newly identified OBI cases were more frequent in men than in women, which is in agreement with the findings of Saha et al. in India (Saha et al., 2017). Differences in disease susceptibility between men and women have been reported in other diseases as well and might be attributed to differences in behavior and biology. For example, women have been shown to possess a stronger immune response against HBV (Brown et al., 2022) and renal cancer patients (Ning et al., 2023). In the current study, there was no difference in CD4+ T-cell counts or HIV viral load between OBI^+^ and OBI^−^ participants at baseline. These findings align with the results reported previously (Ryan et al., 2017; Phinius et al., 2023). Newly identified OBI cases, however, had higher HIV viral loads at year 1 than OBI-negative participants. After 1 year of follow-up, OBI-positive participants had an increased CD4+ T-cell count compared to a decrease in CD4+ T-cell count in OBI-negative participants (*p* = 0.01), Table 5 and Supplementary Figure S3b. This trend was also seen in anti-HBc-positive cases compared to anti-HBc-negative cases. These latter results were unexpected as a previous study associated anti-HBc with poor HIV control during HIV treatment (Malagnino et al., 2023). However, the relatively low numbers make it difficult to reach any firm conclusions.

There was no statistically significant difference between HBV-negative, HBsAg-positive, and OBI-positive participants for all variables tested at baseline except ALT. These results should be interpreted with caution as ALT results were available for only four of the eight HBsAg-positive participants. Other studies including one in Botswana also reported no difference between HBV-negative, HBsAg-positive, and OBI-positive participants, including in the non-invasive markers of liver damage, APRI and FIB-4 (Ryan et al., 2017; Mbangiwa et al., 2018; Phinius et al., 2020).

The 2024 HBV guidelines recommend that newly diagnosed PWH should be screened for *HBsAg, anti-HBs, and anti-HBc*. It also mentions OBI under management considerations for special populations. It further recommends pre-emptive therapy if there is a risk for HBV reactivation such as during immunosuppressive therapy (see footnote 2). Data from this study can be used to guide the identification of participants at risk of HBV reactivation. HBV DNA screening should be considered in PWH before initiating therapy with no anti-HBV active drugs, especially in anti-HBc-positive participants. Further research on the kinetics of new biomarkers such as HBV RNA levels, and HBV-related antigens should be explored in OBI participants as biomarker profiles in OBI are still not clear.

The limitations of this study are that participants in the natural HIV cohort had relatively high CD4+ T-cell counts; thus, the cohort does not represent OBI dynamics in PWH with low CD4+ T-cell counts. Furthermore, HIV duration of infection was unknown as HIV recency was not tested for in this study. Some participants did not have available/sufficient samples for the various tests conducted at all time points, resulting in 90 participants with OBI results for at least two time points being used to estimate the newly identified OBI rate. However, participant demographics were the same between those in the OBI estimation cohort and the rest of the cohort. In addition, because of insufficient sample volumes, anti-HB titers were not measured. The short duration of follow-up may explain the lack of significant differences in several of the analyses performed in this study. This study included only young participants (younger than 40 years old); however, other studies in Botswana which included both young and old participants also reported high OBI prevalence, which was nevertheless not significantly associated with age (Ryan et al., 2017; Phinius et al., 2023). However, this study is invaluable in that it provides OBI natural disease dynamics in a population, which cannot be replicated easily in the HIV test-and-treat era.

## 5 Conclusion

This study reported a high prevalence of possible reactivations and persistence of OBI among treatment-naive young PWH in Botswana. Newly identified OBI was more common in men and in participants with lower CD4^+^ T-cell counts. There were possible HBV reactivations with the majority of cases being anti-HBc seropositive OBIs. A proportion of anti-HBc^−^ OBIs was due to possible reactivations with loss of anti-HBc. There were minimal OBIs resulting from HBsAg loss and minimal possible reactivations resulting in HBsAg positivity. OBI screening in PWH should be considered because of the risk of transmission and possible reactivation of HBV from these individuals.

## Data availability statement

The raw data supporting the conclusions of this article will be made available by the authors, without undue reservation.

## Ethics statement

The studies involving humans were approved by Health Research Development Committee (HRDC) at the Botswana Ministry of Health [Ethics permit number: PPME 13/1811V(318)]. The studies were conducted in accordance with the local legislation and institutional requirements. The participants provided their written informed consent to participate in this study.

## Author contributions

MA: Conceptualization, Data curation, Formal analysis, Funding acquisition, Investigation, Methodology, Project administration, Supervision, Validation, Visualization, Writing – original draft, Writing – review & editing. BBP: Data curation, Formal analysis, Methodology, Supervision, Validation, Visualization, Writing – review & editing. BKP: Data curation, Formal analysis, Investigation, Visualization, Writing – review & editing. MM: Formal analysis, Methodology, Validation, Visualization, Writing – review & editing. LB: Data curation, Investigation, Methodology, Supervision, Visualization, Writing – review & editing. GNT: Data curation, Investigation, Validation, Writing – review & editing. PM: Data curation, Formal analysis, Investigation, Methodology, Writing – review & editing. TR: Data curation, Investigation, Visualization, Writing – review & editing. KB: Data curation, Investigation, Validation, Visualization, Writing – review & editing. GM: Data curation, Investigation, Visualization, Writing – review & editing. WC: Data curation, Formal analysis, Methodology, Validation, Visualization, Writing – review & editing. RM: Resources, Supervision, Visualization, Writing – review & editing. DG: Conceptualization, Supervision, Visualization, Writing – review & editing. JB: Supervision, Validation, Visualization, Writing – review & editing. SM: Formal analysis, Methodology, Resources, Supervision, Validation, Visualization, Writing – review & editing. AK: Supervision, Validation, Visualization, Writing – review & editing. SG: Conceptualization, Formal analysis, Funding acquisition, Methodology, Project administration, Resources, Supervision, Validation, Visualization, Writing – review & editing.

## Footnotes

^1^ https://iris.who.int/bitstream/handle/10665/376353/9789240090903-eng.pdf?sequence=1
