# Supplementary figures and images for: Persistence and risk factors of occult hepatitis B virus infections among antiretroviral therapy-naïve people living with HIV in Botswana
Source: Front Microbiol. 2024 May 9;15:1342862. doi: 10.3389/fmicb.2024.1342862 (PMC11112038; doi:10.3389/fmicb.2024.1342862)

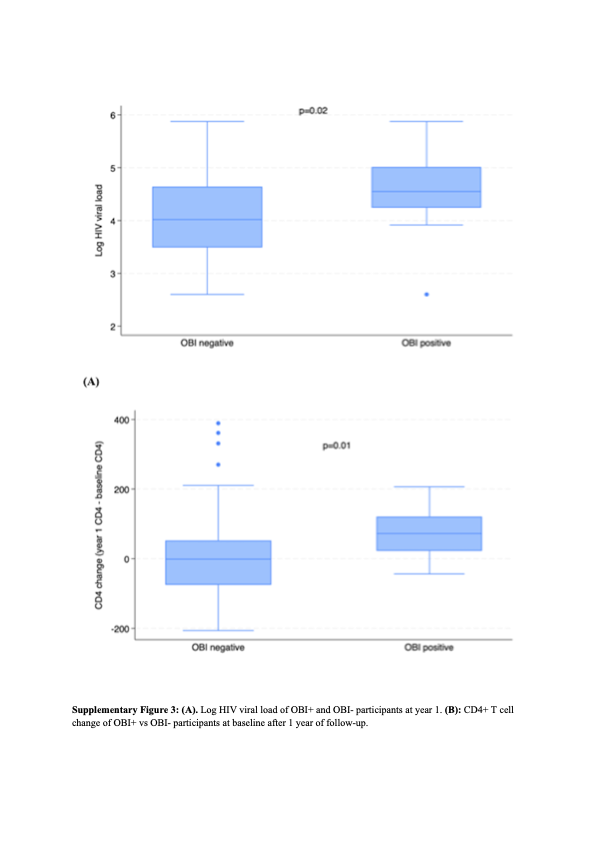

Supplement: Supplementary file 1 [file Data_Sheet_1.ZIP › Figures_24012024-UPDATED/Supplementary Figure 3_24012024.tiff]

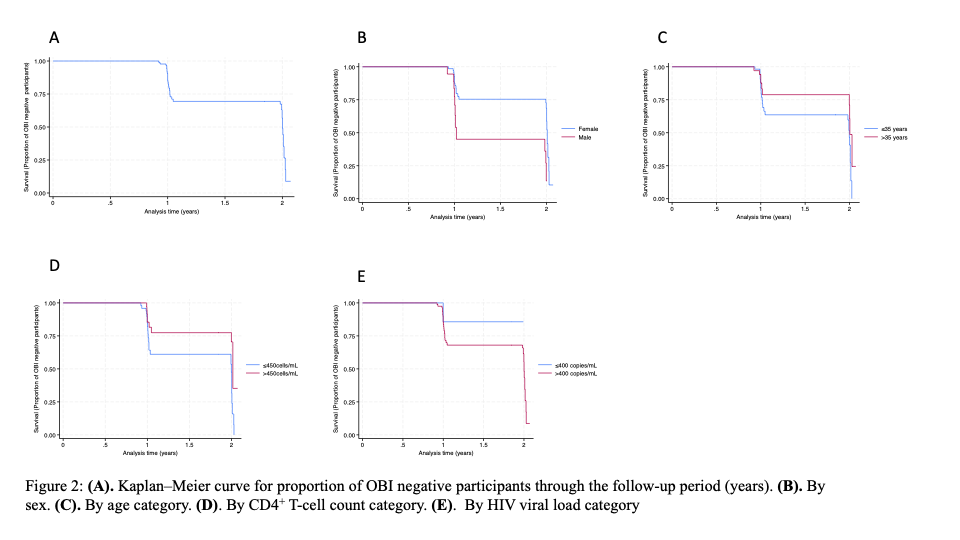

Supplement: Supplementary file 1 [file Data_Sheet_1.ZIP › Figures_24012024-UPDATED/Figure 2_23012024.tiff]

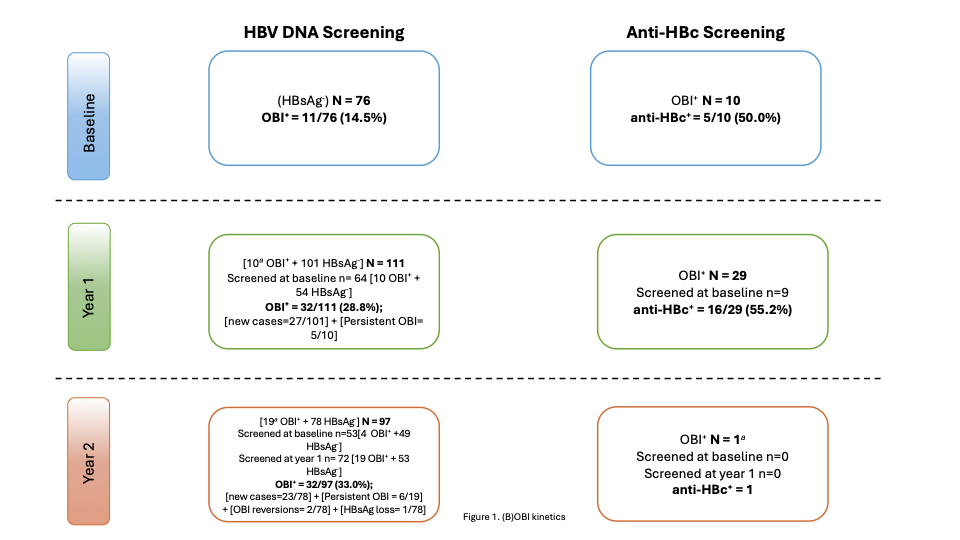

Supplement: Supplementary file 1 [file Data_Sheet_1.ZIP › Figures_24012024-UPDATED/Figure 1B_24012024.tiff]

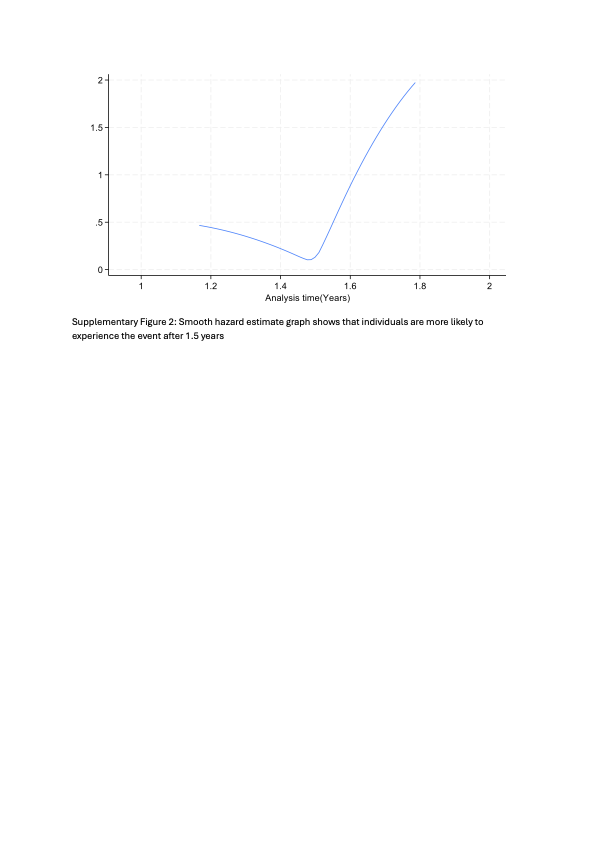

Supplement: Supplementary file 1 [file Data_Sheet_1.ZIP › Figures_24012024-UPDATED/Supplementary figure 2_23012024.tiff]

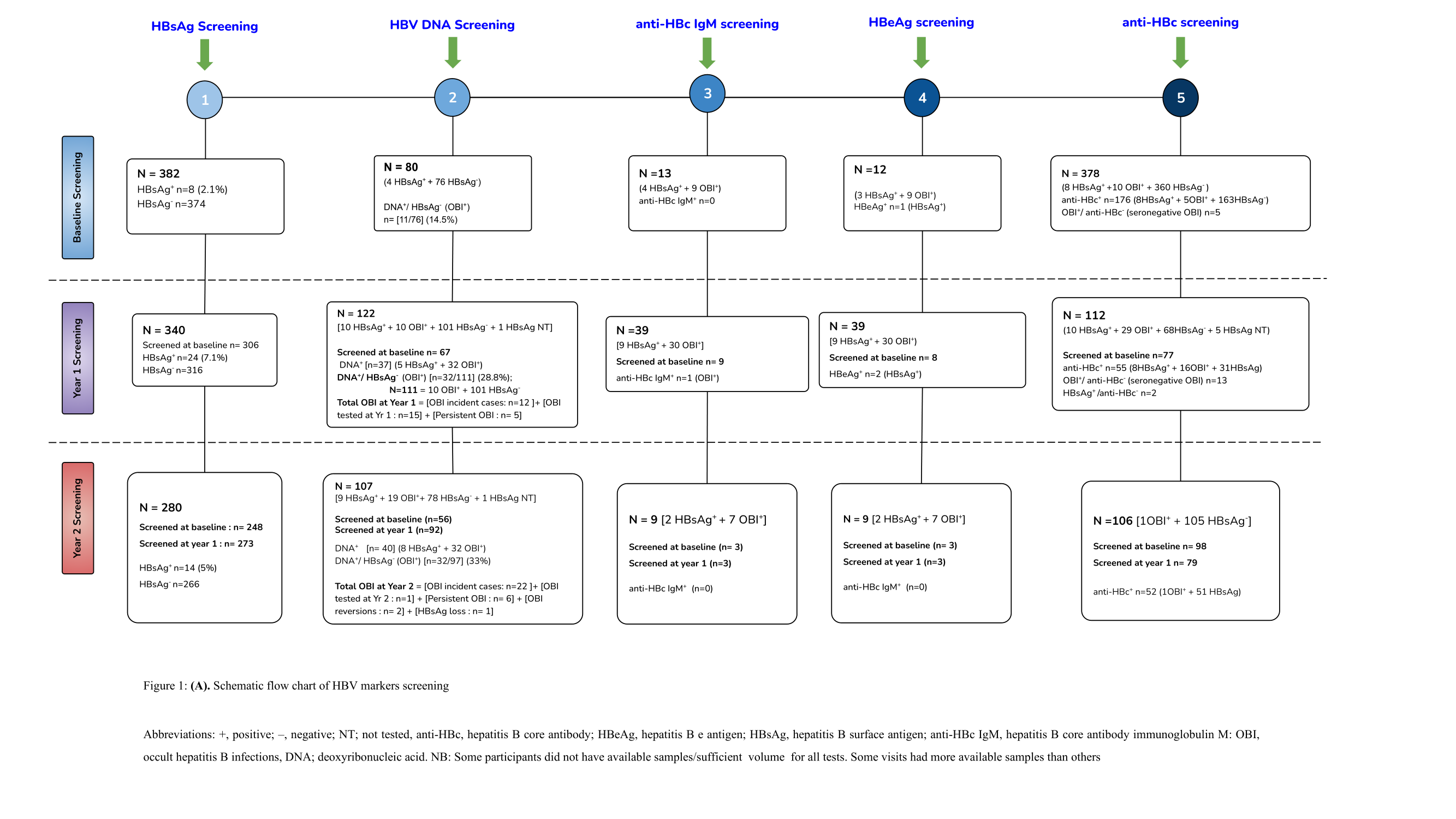

Supplement: Supplementary file 1 [file Data_Sheet_1.ZIP › Figures_24012024-UPDATED/Figure 1A-Updated 24012024.tiff]

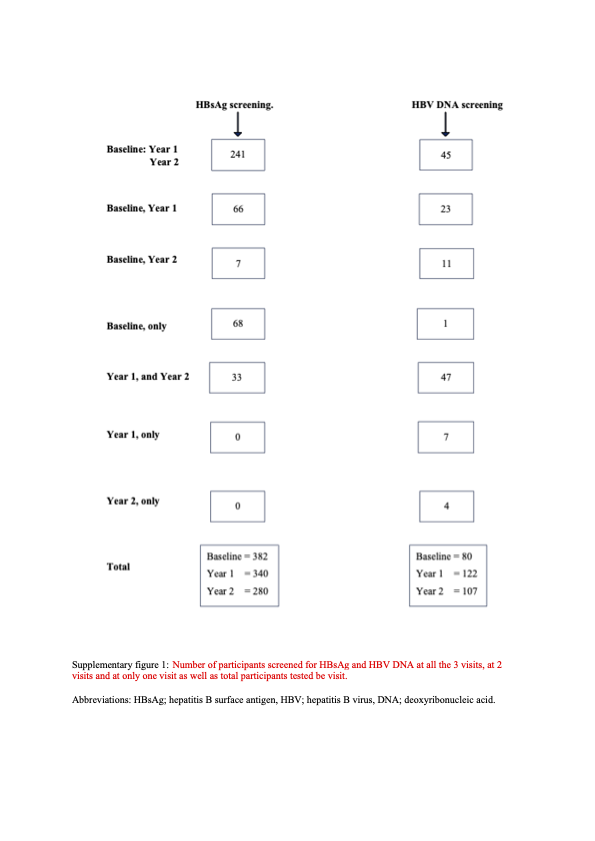

Supplement: Supplementary file 1 [file Data_Sheet_1.ZIP › Figures_24012024-UPDATED/Supplementary figure 1_24012024.tiff]
