# Supplementary material for: Persistence and risk factors of occult hepatitis B virus infections among antiretroviral therapy-naïve people living with HIV in Botswana
Source: Front Microbiol. 2024 May 9;15:1342862. doi: 10.3389/fmicb.2024.1342862 (PMC11112038; doi:10.3389/fmicb.2024.1342862)
Supplement: Supplementary file 2 [file Table_1.doc]

Supplementary table 1: Cross sectional CD4 and VL by anti-HBc status

| **Outcome** | **Anti-HBc negative** | **Anti-HBc positive** | **P-value** |
| --- | --- | --- | --- |
| CD4+ T-cell (baseline), n = 82 | 372 (319 – 505) | 429 (308 – 536) | 0.3 |
| CD4+ T-cell (year 1), n = 56 | 366 (290 – 500) | 474 (337 – 614) | 0.03 |
| Log (10) HIV VL (baseline), n = 83 | 4.10 (3.82 – 4.70) | 4.22 (3.77 – 4.97) | 0.4 |
| Log (10) HIV VL (year 1), n=79 | 4.30 (3.80 – 4.77) | 4.16 (3.56 – 4.64) | 0.6 |

Anti-HBc, Hepatitis B virus core antibodies; HIV: human immunodeficiency virus, VL: viral load.
